# Correlation between the biomechanical characteristics and stability of the 143D movement during the balance phase in competitive Tai Chi

**DOI:** 10.3389/fbioe.2024.1449073

**Published:** 2024-10-09

**Authors:** Ruifeng Huang, Yong Ma, Shijie Lin, Weitao Zheng, Lin Liu, Mengyao Jia

**Affiliations:** ^1^ Engineering Research Center of Sports Health Intelligent Equipment of Hubei Province, Wuhan Sports University, Wuhan, China; ^2^ Research Center of Sports Equipment Engineering Technology of Hubei Province, Wuhan Sports University, Wuhan, China; ^3^ Key Laboratory of Sports Engineering of General Administration of Sports of China, Wuhan Sports University, Wuhan, China; ^4^ Department of Physical Education, Intelligent Sports Engineering Research Center, Northwest Polytechnical University, Xi’an, China

**Keywords:** joint angle, joint moment, integrated electromyography, root mean-squared amplitude, stability index

## Abstract

**Objective:**

To explore the biomechanical factors affecting the stability of athletes in the 143D balance phase of competitive Tai Chi.

**Method:**

The Vicon 3D motion capture system, Kistler 3D force platform, and Noraxon surface electromyography (sEMG) system were used to measure the joint angle, joint moment, center of gravity, ground reaction force, and sEMG data of athletes. The stability index was then calculated according to the formula. Pearson’s or Spearman’s correlation tests were used to analyze the associations between the biomechanical factors and stability index.

**Results:**

(1) Medial lateral stability index (MLSI): A significant negative correlation was found between the ankle inversion angle of the supporting leg (SL) and MLSI (*p* < 0.05). (2) Anterior posterior stability index (APSI): Significant negative correlations were observed between the ankle intorsion angle, integrated electromyography (iEMG) of the gastrocnemius, and muscle contribution rates of the tibialis anterior, external oblique, and gastrocnemius of the non-supporting leg (NL) with the APSI (*p* < 0.05). The ankle dorsiflexion moment, iEMG of the rectus femoris and tibialis anterior, muscle contribution rate of the biceps femoris, and root mean-squared (RMS) amplitude of the gluteus maximus of the SL also showed significant negative correlations with the APSI (*p* < 0.05). Strong and significant negative correlations were also identified between the hip intorsion angle, iEMG of the tibialis anterior, and RMS amplitude of the rectus femoris of the NL with the APSI (*p* < 0.01). Further strong and significant negative correlation was also found between the RMS amplitude of the biceps femoris of the SL and APSI (*p* < 0.01). The knee extorsion angle of the NL was positively correlated with the APSI (*p* < 0.05). (3) Dynamic postural stability index (DPSI): The knee adduction angle, iEMG of the tibialis anterior, and RMS amplitude of the erector spinae of the NL were significantly positively correlated with the DPSI (*p* < 0.05). The knee abduction and hip extension moments of the SL were also significantly positively correlated with the DPSI (*p* < 0.05).

**Conclusion:**

The ankle inversion angle of the SL impacts left–right stability, while the NL’s hip and ankle intorsion angles, knee extorsion angle, and exertion on the core muscle and SL’s main muscles, as well as exertion of specific muscles of the NL affect anterior–posterior stability. The hip extension and knee abduction moments of the SL, knee adduction angle, exertion on the tibialis anterior, and activation of the erector spinae of the NL significantly affect the overall stability of an athlete.

## 1 Introduction

Tai Chi is one of the most important traditional Chinese martial arts. It is characterized by its principles of using softness to overcome hardness, neutralizing and issuing energy, and its philosophical emphasis on harmonious integration (Yang et al., 2022; Yang and Yang, 2017). Practicing Tai Chi has been shown to promote physical health (Gatts and Woollacott, 2007). Since the introduction of the Martial Arts Routine Competition Regulation (MARCR) in 1958, Tai Chi has evolved into a competitive martial art form known as competitive Tai Chi (CTC). CTC is a sports event that features continuous, complex, and high-intensity Tai Chi movements performed to music. In recent years, given the continuous updates to the MARCR and improvement of CTC levels, the inclusion of designated and innovative difficult-level movements has driven CTC toward more advanced, newer, more intricate, and more exquisite practices (Wang and Qiu, 2012).

The difficulty levels of the balance movements in CTC are categorized into four types as A, B, C, and D, with the difficulty increasing gradually from A to D. In the latest MARCR issued in 2023, the code number of the low-balance with cross of back leg movement was assigned as 143D, indicating that this is the most challenging and also one of the highest scoring balance movements. Athletes frequently incorporate the 143D movement in CTC because it helps improve their competition scores and is crucial for connecting the one movement to the next (Sun, 2023). According to MARCR, when performing the 143D movement, CTC athletes can only use one of their legs as the supporting leg (SL) to execute the single-leg squat. The balance phase of the 143D movement begins when the athlete squats to the point where the thigh of the SL is no higher than horizontal, while the non-supporting leg (NL) is crossed behind the SL. During this phase, the NL is prohibited from touching the ground. Research indicates that whole-body stability significantly impacts the sports performances of CTC athletes, and point deductions are commonly applied as penalties during the phase that requires maintaining balance (Yi et al., 2019). Muscle exertion and joint biomechanics are critical factors that affect the stability of the athlete and also represent the ability of the athlete to maintain posture control (Gribble and Robinson, 2009; Borghuis et al., 2011; Brough et al., 2021).

The dynamic postural stability index (DPSI) is an effective method of evaluating body stability through calculation of the ground reaction force (GRF); it was first proposed by Wikstrom et al. (2005) and can be used to evaluate not only the posture control abilities of patients with functional ankle instabilities (Brown et al., 2010) but also the balancing abilities of athletes practicing different sports (Frisch et al., 2011). Previous studies on other types of sports have demonstrated that biomechanical indicators significantly impact postural stability (Booysen et al., 2015; Robinson and Gribble, 2008; Borghuis et al., 2011). However, there is currently limited research on the impacts of the biomechanical indicators on the postural stabilities of CTC athletes when performing movements from A to D levels. Further investigations are thus required on the posture control and muscle exertion strategies of CTC athletes during execution of the 143D movements. Therefore, the Vicon motion capture system, Kistler force platform, and Noraxon surface electromyography (sEMG) system were used in this study to analyze the correlations between the kinematic, kinetic, and sEMG characteristics as well as DPSI during the balance phase of the CTC 143D movements. The present work aims to deepen the understanding regarding the movement characteristics and balance mechanisms of the 143D movements by providing theoretical support for improving the training effectiveness and sports performance in CTC.

## 2 Methods

### 2.1 Participants

G Power 3.1.9.2 was used in this study to calculate the predicted sample size. A larger effect size of 0.8 was set for the correlation analysis, and at least nine subjects are required with an *α* of 0.05 and a power of 0.8 (Kang, 2021). Thus, 10 male athletes at or above the first level of CTC were recruited for this study from Hubei Province. All subjects have participated in at least provincial-level CTC events and their bodies are in good health; further, they did not have any conditions affecting their sports performance.

Among the participants, three athletes were at the elite level while the remaining seven were first-level athletes; the average age of the participants was 19.00 ± 0.81 years, average height was 177.50 ± 3.75 cm, average bodyweight was 66.30 ± 2.45 kg, and average training period was 9.71 ± 2.43 years. The dominant side of the lower limbs of all subjects was the right side. All participants were informed of the experimental protocols and signed the informed consent form. The study was approved by the Wuhan Sports University Medical Ethics Committee (number 2023076).

### 2.2 Study design

#### 2.2.1 Preparation

The subjects ran slowly on a treadmill at a speed of 5 m/s and a slope of 0% for 5 min as the warm-up session, followed by stretching for 5 min based on their pretraining routines. Before testing, all participants underwent warm-up running sessions to enable them to complete the 143D movement efficiently. They practiced the 143D movement in advance so as to adapt to the position and size of the force table in the laboratory and sat quietly for 2 min. Then, sEMG electrodes were attached to subjects as follows: the skin surface was first disinfected with alcohol; then, two electrodes were applied at the belly muscle; next, the Noraxon wireless sEMG collector was applied and secured with muscle tape. Because the 143D is a single-leg squat balance movement, the main muscles of the lower limbs and core muscles were the main targets in this study. The names and positions of the muscles monitored in the study are shown in Table 1.

**TABLE 1:** Names and positions of the muscles monitored by sEMG.

Position	Muscle	Abbreviation
Bilateral lower limbs	Rectus femoris	RF
	Tibialis anterior	TA
	Biceps femoris	BF
	Lateral gastrocnemius	GA
	Gluteus maximus	GM
Both sides of the torso	External oblique	EO
	Erector spinae	ES

**FIGURE 1:**
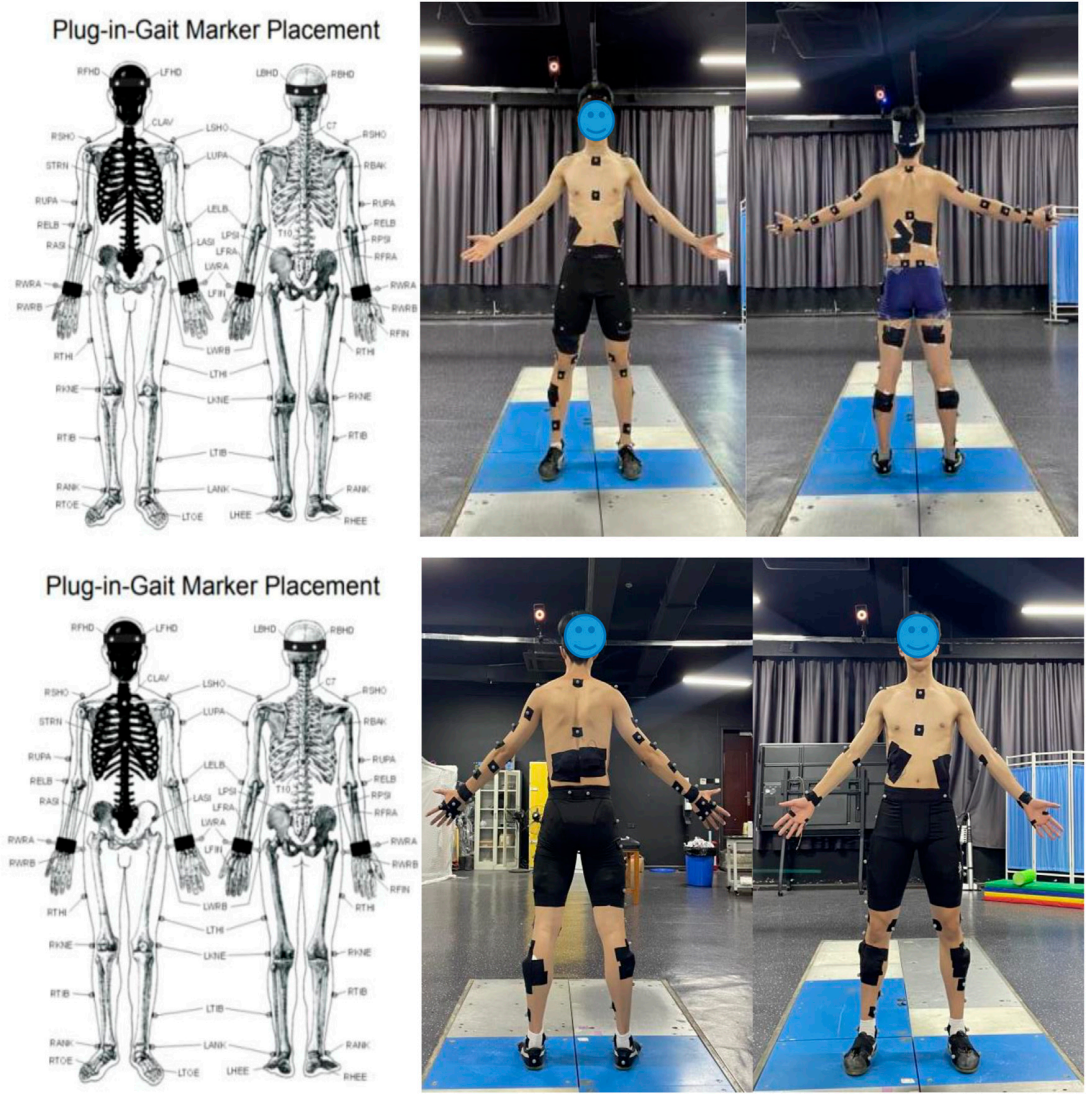
Scheme showing the placement of the reflective markers.

Reflective markers were then applied to the bodies of the athletes. According to the plug-in-gait marker placement labeling scheme in the Vicon technical manual, at least 39 markers are required. Given the characteristics of Tai Chi movements and the need to analyze the indicators, the number of reflective markers was increased to 70, as shown in Figure 1.

#### 2.2.2 Procedure

According to the sEMG operation manual, the maximum voluntary contraction (MVC) of the muscles was measured using the Noraxon wireless sEMG collector (Ultium EMG, Noraxon, United States, sampling frequency of 1,500 Hz). The subjects were instructed to perform MVC for 5 s, with three tests per muscle and a 60 s rest interval between each test.

The Vicon 3D motion capture system was used in this study for kinematic data collection using nine infrared cameras (T40, Vicon, UK, sampling frequency of 250 Hz). The kinetic data were collected using a Kistler 3D force platform (9260AA6, Switzerland, sampling frequency of 1,500 Hz). The sEMG signals were recorded from the lower limbs and core muscles of the subjects using the Noraxon system. Initially, the subjects stood on two force platforms on both feet in an anatomical position and maintained this posture for 2 s before performing the 143D movements according to the competition routines. The data were successfully collected at least three times from each athlete. The success criterion for the 143D movement is as follows: an on-site professional referee awards a full score, meaning that the subjects must maintain the balanced posture for 2 s during the 143D balance phase. Additionally, the trials were considered successful if there were no noticeable losses from the markers and the sEMG signals were collected without issues. The trials that did not meet these criteria were deemed unsuccessful, and all failed trials were recorded as well.

### 2.3 Indicators

**FIGURE 2:**
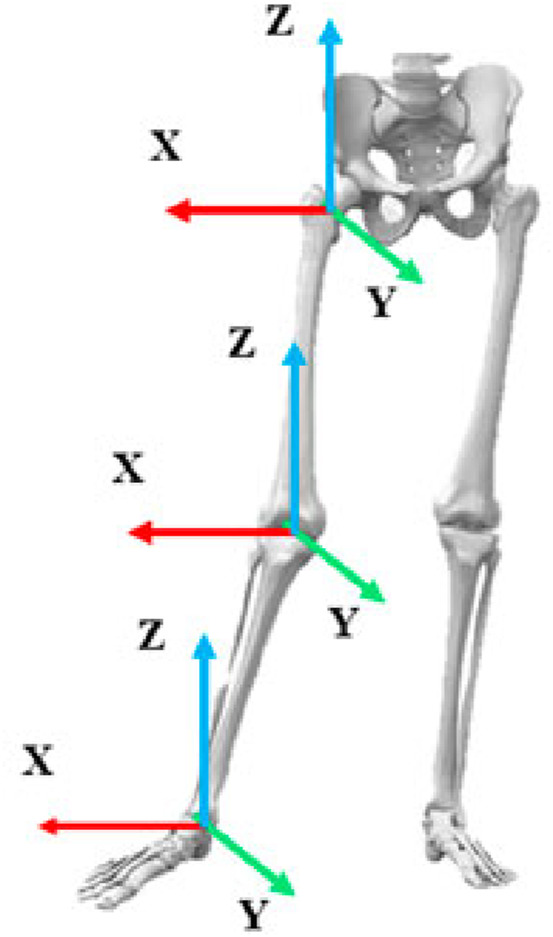
Schematic diagram of the joints and coordinate systems.

Based on the characteristics of the 143D movement, biomechanical research on difficult-level movements in CTC, and biomechanical research on balance movements in other sports events (Zhao et al., 2020; Li et al., 2020; Ma et al., 2016), the knee, ankle, and hip joint moments (Nm·kg^-1^) were selected as the kinetic indicators along with the elevation of the human center of gravity (COG; m) as well as hip, knee, and ankle joint angles (°). The joint coordinate system used for the calculations is shown in Figure 2. The flexion and extension movements are performed around the *X*-axis, while adduction and abduction movements are considered around the *Y*-axis, and intorsion and extorsion movements are considered around the *Z*-axis.

The sEMG indicators include integrated electromyography (iEMG) (%·s) and root mean-squared (RMS) (%) values (Zhao et al., 2020; Li et al., 2020). The stability indexes of the GRFs in the anterior–posterior and medial–lateral directions were calculated using specific formulas (Wikstrom et al., 2005); these indexes serve as one of the indicators for evaluating balance stability in sports performance. The formulas for the different indexes are as follows.

Medial lateral stability index (MLSI): When describing the 143D movement, this index represents the stability in the left-to-right direction of the body. The Equation 1 is as follows.

$$MLSI=\sqrt{\frac{\sum {\left(0-GRF_{x}\right)}^{2}}{NDP}}÷BW,$$ (1)

Anterior posterior stability index (APSI): This refers to the stability of the body in the anterior-to-posterior direction. The Equation 2 is as follows

$$APSI=\sqrt{\frac{\sum {\left(0-GRF_{y}\right)}^{2}}{NDP}}÷BW.$$ (2)

Dynamic postural stability index (DPSI): This refers to the overall stability of the body. The Equation 3 is as follows

$$DPSI=\sqrt{\frac{\sum {\left(0-GRF_{x}\right)}^{2}+\sum {\left(0-GRF_{y}\right)}^{2}+\sum {\left(BW-GRF_{z}\right)}^{2}}{NDP}}÷BW.$$ (3)

Here, *NDP* refers to the total number of frames recorded in the Vicon motion capture system;

*BW* is the bodyweight;

*GRF*_*x*_, *GRF*_*y*_, and *GRF*_*z*_ are the GRFs in the anterior–posterior, medial–lateral, and vertical directions, respectively.

The larger the values of the MLSI, APSI, and DPSI, the poorer is the balance stability; contrarily, the smaller the values, the better is the balance stability.

### 2.4 Data processing

The coordinates of the markers captured by the Vicon 3D motion capture system were used for modeling. The c3D files were first exported to Visual 3D software for skeleton modeling, following which the kinetic motion files were imported into the static model for matching. The data collected with the 3D force platform were standardized, and the GRF was normalized to a multiple of the bodyweight. The kinetic indicators were calculated through inverse kinetics analysis.

The sEMG data were preprocessed in MR 3.6 software; initially, the three MVCs collected were processed using the Make MVC function, and the best data were selected for further analyses. The 14 processed MVC datasets were combined into a single file, and the channel order of the MVC file was adjusted to match the order in the 143D movement file for subsequent analyses. The 143D movement files were then processed for signal filtering, rectification, smoothing, and MVC normalization to generate a data report, in which the iEMG and RMS values were selected for further analyses.

### 2.5 Event division

In this study, the process of a complete 143D movement is defined as the period from the NL lifting off the ground to the NL touching the ground again. This process is divided into four events and three phases, as shown in Table 2. The focus of this study is on investigating the biomechanical characteristics of the 143D movements in the balance phase.

**TABLE 2:** Event divisions in the 143D movement.

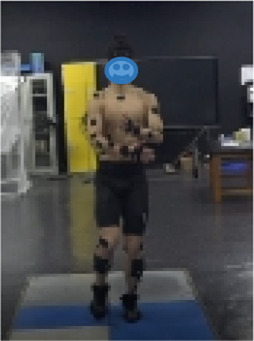	E1→E2	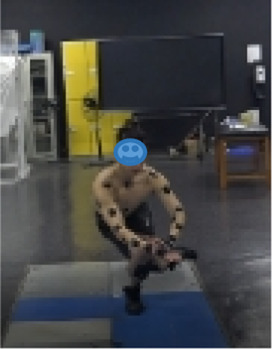	E2→E3	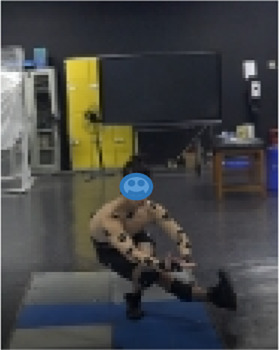	E3→E4	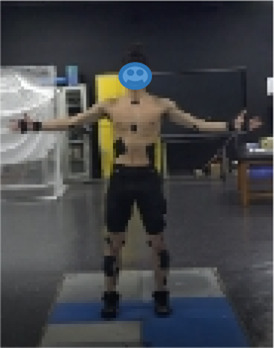
Preparation Moment E1	Squat Phase P1	Balance Moment E2	Balance Phase P2	Retraction Moment E3	Retract Phase P3	Ending Moment E4
When the NL is lifted off the ground		When the knee of the NL starts to undergo extension		When the knee of the NL starts to undergo flexion		When the NL is placed on the ground

### 2.6 Statistical analysis

The Shapiro–Wilk test was conducted using IBM SPSS statistics 25 (IBM Corporation, Armonk, New York, United States) to assess the normality of the data distribution. The outliers were removed, and the Spearman or Pearson correlation test was applied accordingly thereafter to analyze the correlations between the biomechanical indicators and stability indexes. A *p-*value <0.05 was considered a significant correlation between the datasets. Moreover, the strength of the data correlation was categorized based on the magnitude of the correlation coefficient; a correlation coefficient between 0.8 and 1 is considered a robust correlation, and a value between 0.6 and 0.8 is considered a strong correlation (Salkind and Frey, 2019).

## 3 Results

### 3.1 Correlations between the kinematic indicators and stability indexes

According to Table 3, the MLSI of the 143D movement is significantly positively correlated with the adduction angle of the ankle joint in the SL (*p* < 0.05, *r* = 0.681). The APSI is significantly positively correlated with the intorsion angles of the ankle joint in the NL (*p* < 0.05, *r* = 0.692) and hip joint in the NL (*p* < 0.01, *r* = 0.817). Conversely, the extorsion angle of the knee joint in the NL is significantly negatively correlated with the APSI (*p* < 0.01, *r* = −0.824). The DPSI is significantly negatively correlated with the abduction angle of the knee joint in the NL (*p* < 0.05, *r* = −0.697).

**TABLE 3:** Correlations between the kinematic indicators and stability indexes.

Indicators		MLSI		APSI		DPSI	
		CC	Sig (two-tailed)	CC	Sig (two-tailed)	CC	Sig (two-tailed)
NL	KJA-X	−0.108	0.782	−0.328	0.389	0.242	0.531
	KJA-Y	−0.318	0.404	0.401	0.285	−.697*	0.037
	KJA-Z	0.022	0.956	−0.824**	0.006	0.298	0.437
	AJA-X	0.427	0.326	0.329	0.387	−0.228	0.555
	AJA-Y	0.469	0.203	0.692*	0.039	−0.258	0.503
	AJA-Z	−0.583	0.099	−0.191	0.622	0.395	0.293
	HJA-X	−0.431	0.247	−0.092	0.814	−0.007	0.986
	HJA-Y	−0.204	0.598	−0.102	0.793	−0.328	0.393
	HJA-Z	0.231	0.550	0.817**	0.007	−0.418	0.263
COG	COG-X	−0.333	0.381	0.033	0.932	0.421	0.286
	COG-Y	0.512	0.170	−0.301	0.433	0.067	0.865
	COG-Z	−0.606	0.084	−0.386	0.305	0.398	0.289
SL	KJA-X	−0.367	0.331	−0.012	0.975	−0.164	0.674
	KJA-Y	0.163	0.675	0.281	0.464	−0.006	0.987
	KJA-Z	0.536	0.137	−0.236	0.542	0.271	0.482
	AJA-X	0.434	0.243	−0.121	0.757	0.446	0.229
	AJA-Y	−0.317	0.406	0.352	0.356	−0.483	0.187
	AJA-Z	0.681*	0.043	0.029	0.941	0.111	0.775
	HJA-X	−0.359	0.139	−0.329	0.461	0.401	0.765
	HJA-Y	−0.533	0.342	0.283	0.387	−0.117	0.285
	HJA-Z	−0.608	0.083	−0.206	0.596	0.397	0.291

Notes: * represents *p* < 0.05; ** represents *p* < 0.01; NL, non-supporting leg; SL, supporting leg; KJA, knee joint angle; AJA, ankle joint angle; HJA, hip joint angle; COG, center of gravity; ‘–’ represents a negative correlation; CC, correlation coefficient.

### 3.2 Correlations between the kinetic indicators and stability indexes

According to Table 4, none of the kinetic indicators are significantly associated with the MLSI of the 143D movement. However, the ankle dorsiflexion moment of the SL is significantly positively correlated with the APSI (*p* < 0.05, *r* = 0.717), while the APSI is significantly negatively correlated with the ankle intorsion moment of the SL (*p* < 0.01, *r* = −0.850). Additionally, the knee abduction moment (*p* < 0.05, *r* = −0.709) and the hip extension moment (*p* < 0.05, r = −0.726) of the SL are both significantly negatively correlated with the DPSI.

**TABLE 4:** Correlations between the kinetic indicators and stability indexes.

Indicators		MLSI			APSI			DPSI	
		CC		Sig (two-tailed)	CC	Sig (two-tailed)		CC	Sig (two-tailed)
NL	KJM-X	−0.531		0.142	−0.205	0.597		0.354	0.351
	KJM-Y	0.215		0.579	−0.583	0.101		0.277	0.473
	KJM-Z	0.105		0.789	−0.267	0.487		0.388	0.303
	AJM-X	−0.611		0.081	−0.182	0.639		0.261	0.499
	AJM-Y	−0.639		0.064	0.026	0.947		0.055	0.89
	AJM-Z	−0.137		0.726	0.469	0.203		−0.484	0.188
	HJM-X	−0.532		0.141	−0.197	0.611		0.581	0.113
	HJM-Y	0.184		0.636	−0.035	0.929		−0.179	0.644
	HJM-Z	0.141		0.717	0.189	0.627		−0.109	0.781
SL	KJM-X	−0.281		0.464	−0.131	0.737		0.64	0.063
	KJM-Y	0.073		0.853	0.135	0.732		−0.708*	0.033
	KJM-Z	−0.117		0.765	0.317	0.406		−0.133	0.732
	AJM-X	−0.050		0.898	0.717*	0.031		−0.466	0.207
	AJM-Y	0.172		0.659	−0.850**	0.004		0.646	0.061
	AJM-Z	−0.347		0.360	0.181	0.641		−0.16	0.683
	HJM-X	0.424		0.256	0.547	0.127		−0.726*	0.027
	HJM-Y	0.094		0.809	−0.057	0.885		−0.539	0.134
	HJM-Z	0.399		0.287	−0.385	0.306		0.429	0.252

Notes: * represents *p* < 0.05; ** represents *p* < 0.01; NL, non-supporting leg; SL, supporting leg; KJM, knee joint moment; AJM, ankle joint moment; HJM, hip joint moment; ‘–’ represents a negative correlation; CC, correlation coefficient.

### 3.3 Correlations between the iEMG and stability indexes

As shown in Table 5, there are no significant positive correlations with the MLSI of the 143D movement. The APSI is significantly positively correlated with the iEMG values of the RF of the SL (*p* < 0.05, *r* = 0.783), TA of the NL (*p* < 0.01, *r* = 0.812), TA of the SL (*p* < 0.05, *r* = 0.672), and GA of the NL (*p* < 0.05, *r* = 0.758). The DPSI is significantly negatively correlated with the iEMG value of only the TA of the NL (*p* < 0.01, *r* = −0.779).

**TABLE 5:** Correlations between the iEMG values and stability indexes.

iEMG		MLSI		APSI		DPSI	
		CC	Sig (two-tailed)	CC	Sig (two-tailed)	CC	Sig (two-tailed)
NL	RF	0.517	0.154	−0.117	0.765	−0.033	0.932
	TA	0.488	0.182	0.812**	0.008	−0.779*	0.013
	ES	0.238	0.538	0.042	0.915	−0.589	0.095
	GM	0.134	0.732	−0.393	0.295	−0.27	0.482
	BF	0.113	0.772	−0.154	0.691	0.296	0.441
	GA	0.335	0.379	0.758*	0.018	−0.254	0.513
	EO	0.629	0.069	0.439	0.238	−0.067	0.864
SL	RF	0.475	0.196	0.783*	0.013	−0.299	0.435
	TA	−0.43	0.248	0.672*	0.047	−0.442	0.234
	ES	0.07	0.859	−0.351	0.356	−0.179	0.644
	GM	0.327	0.432	0.552	0.156	−0.605	0.112
	BF	0.608	0.083	0.605	0.084	−0.218	0.573
	GA	0.188	0.628	−0.156	0.688	−0.29	0.449
	EO	0.516	0.155	−0.009	0.982	0.19	0.624

Notes: * represents *p* < 0.05; ** represents *p* < 0.01; NL, non-supporting leg; SL, supporting leg; ‘–’ represents a negative correlation; CC, correlation coefficient.

### 3.4 Correlations between the muscle contribution rates and stability indexes

As shown in Table 6, the APSI of the 143D movement is significantly positively correlated with the muscle contribution rates of the TA of the NL (*p* < 0.05, *r* = 0.682), BF of the SL (*p* < 0.05, *r* = 0.750), and GA of the NL (*p* < 0.05, *r* = 0.732). In contrast, the muscle contribution rate of the EO in the NL is significantly negatively correlated with the APSI of the 143D movement (*p* < 0.05, *r* = −0.777). The muscle contribution rates on either side are not significantly correlated with the MLSI of the 143D movements.

**TABLE 6:** Correlations between the muscle contribution rates and stability indexes.

Muscle contribution rate		MLSI		APSI		DPSI		
		CC	Sig (two-tailed)	CC	Sig (two-tailed)	CC	Sig (two-tailed)	
NL	RF	0.544	0.163	0.012	0.977	0.481	0.228	
	TA	0.394	0.294	0.682*	0.043	−0.653	0.057	
	ES	−0.206	0.595	0.345	0.363	−0.521	0.151	
	GM	0.043	0.913	−0.556	0.122	−0.017	0.966	
	BF	−0.001	0.999	−0.151	0.699	0.404	0.281	
	GA	0.147	0.706	0.732*	0.025	−0.208	0.591	
	EO	0.486	0.185	0.032	0.934	0.204	0.598	
SL	RF	−0.008	0.985	0.601	0.087	−0.121	0.757	
	TA	−0.591	0.123	0.432	0.285	−0.206	0.624	
	ES	−0.231	0.549	−0.398	0.289	−0.209	0.589	
	GM	−0.057	0.904	0.469	0.288	−0.322	0.481	
	BF	0.017	0.966	0.750*	0.022	−0.567	0.112	
	GA	−0.011	0.979	−0.211	0.586	−0.224	0.562	
	EO	0.182	0.699	−0.777*	0.041	0.597	0.157	

Notes: * represents *p* < 0.05; ** represents *p* < 0.01; NL, non-supporting leg; SL, supporting leg; ‘–’ represents a negative correlation; CC, correlation coefficient.

### 3.5 Correlations between the RMS values and stability indexes

As shown in Table 7, there are no significant positive correlations with the MLSI of the 143D movement. The APSI is significantly positively correlated with the RMS values of the RF of the SL (*p* < 0.01, *r* = 0.842), GM of the SL (*p* < 0.05, *r* = 0.761), and BF of the SL (*p* < 0.01, *r* = 0.917). The DPSI is significantly negatively correlated with the RMS value of only the ES in the NL (*p* < 0.05, *r* = −0.708).

**TABLE 7:** Correlations between the RMS values and stability indexes.

RMS		MLSI		APSI		DPSI	
		CC	Sig (two-tailed)	CC	Sig (two-tailed)	CC	Sig (two-tailed)
NL	RF	0.346	0.362	−0.076	0.847	0.521	0.151
	TA	0.602	0.086	0.435	0.242	−0.106	0.784
	ES	0.083	0.832	0.151	0.698	−.708*	0.033
	GM	−0.027	0.945	−0.314	0.411	−0.291	0.451
	BF	0.017	0.966	0.321	0.433	0.017	0.966
	GA	0.274	0.475	0.59	0.094	0.011	0.983
	EO	0.532	0.143	0.527	0.144	0.005	0.992
SL	RF	0.262	0.535	0.842**	0.009	−0.058	0.889
	TA	−0.507	0.163	0.642	0.062	−0.264	0.491
	ES	−0.213	0.583	−0.431	0.247	−0.036	0.931
	GM	0.048	0.911	0.761*	0.028	−0.616	0.103
	BF	−0.112	0.798	0.917**	0.001	−0.517	0.154
	GA	0.013	0.974	−0.503	0.168	0.233	0.544
	EO	0.328	0.389	0.153	0.695	0.221	0.568

Notes: * represents *p* < 0.05; ** represents *p* < 0.01; NL, non-supporting leg; SL, supporting leg; ‘–’ represents a negative correlation; CC, correlation coefficient.

## 4 Discussion

This study explores the relationships of the kinematic, kinetic, and muscle exertion characteristics with the stability of the 143D movements during the balance phase. The results show a significant negative correlation between the ankle joint adduction angle of the SL and stability in the left–right directions of CTC athletes. The ankle joint intorsion angle of the NL; dorsiflexion moment of the SL; iEMG values of the RF and TA of the SL as well as GA of the NL; muscle contribution rates of the TA, EO, and GA of the NL as well as BF of the SL; and RMS value of the GM of the SL have significant negative correlations with the stability in the anteroposterior direction. Additionally, the hip intorsion angle of the NL, iEMG value of the TA of the NL, and RMS values of the RF and BF of the SL are strongly and significantly negatively correlated with the stability in the anteroposterior direction. Conversely, the knee extorsion angle of the NL is positively correlated with the stability in the anteroposterior direction. The knee adduction angle of the NL; knee abduction and hip extension moments of the SL; iEMG value of the TA of the NL; and RMS value of the ES of the NL are significantly positively correlated with the overall stability of an athlete.

### 4.1 Correlations between the MLSI and biomechanical indicators

During the balance phase of the 143D movement, CTC athletes must maintain the single-leg squat position using only their SLs, while ensuring that their thighs do not rise above the horizontal level. The NL must then be crossed below the thigh of the SL, with the anterior aspect of the thigh of the NL being in contact with the posterior aspect of the thigh of the SL. The NL must be extended maximally, and the ankle joint should be in a dorsiflexed state to prevent the foot from touching the ground to avoid a penalty.

The results reveal a significant negative correlation between the adduction angle of the ankle joint of the SL and MLSI, indicating that a larger ankle adduction angle of the SL is associated with reduced stability in the left–right direction. According to Kibele et al. (2015), the body during balance can be described as a continuously fluctuating inverted pendulum system. An athlete can maintain balance when the vertical projection of the COG of their body remains within the base of support (BoS) (Hof, 2007). An increased adduction angle of the ankle joint of the SL reduces the contact area between the sole and ground. Since the foot of the SL serves as the BoS, a smaller BoS decreases the distance (moment arm) from the vertical line of gravity to both edges of the BoS. This reduction in the moment arm diminishes the stabilizing moment generated by gravity, thereby impairing the ability to resist the tilting moment (Brough et al., 2021). Therefore, it is more challenging for CTC athletes to maintain stability in their left–right directions under these conditions.

### 4.2 Correlations between the APSI and biomechanical indicators

In the 143D movement, maintaining stability during the single-leg squat through the SL is crucial, and the TA of the SL plays a vital role in maintaining the dorsiflexed position of the ankle joint (Harput et al., 2013) supported by the other calf muscle groups to ensure that the COG of the body remains within the BoS (Krkeljas, 2018). Additionally, muscles like the BF and RF of the thigh also help stabilize the knee joint of the SL (Iacono et al., 2016; Booysen et al., 2015).

The plantar flexion of the ankle joint is mainly achieved through concentric contractions of the GA and TA of the NL. However, our results reveal significant negative correlations between the stability in the anteroposterior direction and sEMG characteristics of the TA, GM, RF, and BF of the SL. Similarly, there are significant negative correlations with the sEMG characteristics of the TA and GA of the NL, suggesting that the excessive activities and activation levels of these muscles impair the stability of the athletes in the anteroposterior direction. Although previous studies have demonstrated that co-activation of the thigh muscles during squatting increases knee joint stiffness and maintains stability, the effects of co-activation on stability remain unclear (Kean et al., 2006). Therefore, high-level co-activation may not be an effective strategy as it can unnecessarily stiffen the joints and reduce knee flexion ability (Booysen et al., 2015); this could explain why the activity and activation of the main thigh muscle group in the SL in this study negatively affect stability in the anteroposterior direction.

Moreover, during isometric contraction of the TA of the SL, increased co-activation of the agonist muscle generates a higher net moment from these muscles (Behrens et al., 2015). Consequently, the TA activity level in the SL directly influences the dorsiflexion moment of the ankle joint. An excess dorsiflexion moment can cause the SL to tilt forward, thereby compromising the stability of the athlete in the anteroposterior direction.

This study reveals significant negative correlations of the hip and ankle intorsion angles of the NL with the stability of the athlete in the anteroposterior direction, while the knee extorsion angle is significantly positively correlated with the stability of the athlete in the same direction. To prevent the lateral side of the NL’s foot from touching the ground, the athlete must control the ankle joint of the NL to execute an intorsion movement. According to kinetic chain theory, body movements are transmitted sequentially from the proximal to the distal joints, meaning that the intorsion of the NL’s ankle is influenced by the movements of both the knee and hip joints (Prieske et al., 2015). To avoid the penalty for foot contact with the ground, CTC athletes often increase the hip intorsion angles of the NL, thereby increasing the distance between the lateral side of the foot and the ground. However, this adjustment decreases the knee extorsion angle, leading to overall intorsion of the NL. This overall intorsion of the entire NL toward the lower left contradicts the requirements of the 143D movement, where the NL should remain behind the knee joint of the SL. Consequently, the support force provided by the NL to the SL decreases, requiring the SL to increase muscle exertion to maintain the single-leg squat posture and thereby affecting the athlete’s stability in the anteroposterior direction. Additionally, the TA and GA mainly control the knee and ankle joint movements, which also explains why the force exerted by these muscle groups may negatively impact the stability of the athlete in the anteroposterior direction.

The results also reveal a significant positive correlation between the ankle intorsion moment of the SL and stability of the athlete in the anteroposterior direction, indicating that a smaller ankle intorsion moment of the SL is associated with poorer stability of the athlete in this direction. The intorsion state of the ankle joint, determined by the moment around the vertical axis of the SL, is influenced by both the lower limb muscle groups and position of the NL. Specifically, when the thigh of the NL is positioned on the lower left side of the SL’s knee, it provides a supportive force toward the upper right side. However, as mentioned previously, when the NL rotates inward, the supportive force provided to the SL decreases. In the absence of SL foot movements, the calf may exhibit a counterclockwise rotation tendency, reducing the intorsion moment of the SL ankle joint. Studies on individuals with functional ankle instability (FAI) show that FAI can impair motion control, often forcing the individuals to adopt non-ankle strategies to maintain balance (Wikstrom et al., 2007). Such alterations in an athlete’s motion control strategy can cause changes in technical movements, potentially explaining why the ankle joint moment of the SL and muscle exertion observed in this study are associated with reduced anteroposterior stability.

Lastly, we found that a higher contribution rate from the SL’s EO is correlated with better stability in the anteroposterior direction. When an athlete maintains a single-leg squat position, the distribution of the bodyweight plays a crucial role in stability. Specifically, the lower body contributes more to the anterior aspect of the COG, while the upper body contributes more to the posterior aspect as it is heavier. To counterbalance this, the athlete must tilt their torso forward, shifting the weight toward the front to better align their COG and enhance the anteroposterior stability. The core muscle group, including the EO, is vital in achieving sagittal stability of the upper body during the balance phase (Borghuis et al., 2011). Given that the thigh of the SL must remain below the horizontal line during the squat, the distance between the abdomen and thigh of the SL is shorter when the torso is tilted forward, resulting in uneven distribution of mass on the left and right sides of the body. Behrens et al. (2015) explored the muscle activities in squatting exercises under different load distributions and found that uneven mass distribution of the squatting load places a greater demand on the trunk flexor muscles; this finding is aligned with the observations of this study, where the EO of the SL must be involved more when maintaining a forward tilting posture during the 143D movement.

### 4.3 Correlations between the DPSI and biomechanical indicators

The DPSI is a comprehensive measure of the stability of an athlete in multiple directions (Sell, 2012). We found that higher activity levels of the TA in the NL are associated with improved overall stability in CTC athletes. This finding appears to contradict the earlier results, where increased iEMG values of the TA in the NL were linked to reduced stability in the anteroposterior direction. However, since the DPSI reflects the overall stability, it is suggested that although the higher iEMG value of the TA of the NL may negatively impact stability in the anteroposterior direction, it could still contribute positively to the overall stability. Therefore, athletes should maintain the force exerted by this muscle group during the balance phase of the 143D movement to ensure overall body balance.

A significant positive correlation was observed between the SL’s hip joint extension moment and dynamic posture stability, indicating that a greater hip joint extension moment is related with better dynamic postural stability. Given the closed kinetic chain movement of the SL and the lack of significant impacts of the hip, knee, and ankle flexion–extension angles on dynamic postural stability, these effects may be attributed to changes in the hip joint extension moment caused by the torso, which in turn affects the dynamic postural stability of the body (Norris and Trudelle-Jackson, 2011).

A significant positive correlation was also found between the activation level of the ES of the NL and dynamic posture stability, revealing the importance of core muscle activation in maintaining overall postural stability. This aligns with the findings of Kibler et al. (2006) and Prieske et al. (2015), which suggests that the trunk muscle group achieves a proximal-to-distal force exertion pattern, effectively controlling and protecting the distal joints. Additionally, preactivation of the proximal muscle group may enhance the muscle activation of the limbs, thereby improving the dynamic postural stability. Currently, research on the correlations between knee joint adduction/abduction and dynamic postural stability is limited. However, in the context of the 143D movement, the position of the NL relative to the SL’s knee joint influences the knee adduction angle of the NL. The closer the NL is to the posterior aspect of the knee joint of the SL, the greater is the outward support force exerted on the SL’s knee joint, resulting in a larger abduction moment.

### 4.4 Limitations

This study has some notable limitations. First, the 143D movement is divided into three phases, each of which is very important. Future research will focus on investigating the variations in muscle states across the different phases of the 143D movement using OpenSim. Second, the present study mainly entails the biomechanical factors affecting the stability of the athlete during the 143D balance phase. Hence, there is scope for future research on the biomechanical factors affecting the performances of athletes in the other phases of CTC.

## 5 Conclusion

In summary, the ankle inversion angle of the SL significantly contributes to the stability of the athlete in the left–right direction. The stability in the anteroposterior direction is significantly influenced by the ankle and hip intorsion angles as well as knee extorsion angle of the NL, in addition to exertion on the core muscles, lower limb muscles of the SL, and partial muscle groups of the NL. Additionally, the hip extension and knee abduction moments of the SL, knee adduction angle of the NL, exertion on the TA of the NL, and activation of the ES are critical factors affecting the overall stability of the athlete.

## Data Availability

The original contributions presented in this study are included in the article/Supplementary Material, and any further inquiries may be directed to the corresponding authors.

## Abbreviations

APSI, anterior posterior stability index; BF, biceps femoris; CTC, competitive Tai Chi; DPSI, dynamic postural stability index; EO, external oblique; ES, erector spinae; GA, lateral gastrocnemius; GM, gluteus maximus; GRF, ground reaction force; iEMG, integrated electromyography; MARCR, Martial Arts Routine Competition Regulation; MLSI, medial lateral stability index; MVC, maximum voluntary contraction; NL, non-supporting leg; RF, rectus femoris; RMS, root mean-squared; sEMG, surface electromyography; SL, supporting leg; TA, tibialis anterior.
